# High density of peritumoral lymphatic vessels measured by D2-40/podoplanin and LYVE-1 expression in gastric cancer patients: an excellent prognostic indicator or a false friend?

**DOI:** 10.1007/s10120-012-0216-8

**Published:** 2012-12-14

**Authors:** Julia Rudno-Rudzinska, Wojciech Kielan, Zygmunt Grzebieniak, Piotr Dziegiel, Piotr Donizy, Grzegorz Mazur, Monika Knakiewicz, Ewelina Frejlich, Agnieszka Halon

**Affiliations:** 1 2nd Department of General and Oncological Surgery, Wroclaw Medical University, Borowska 213, 50-556 Wrocław, Poland; 2 Department of Histology and Embryology, Wroclaw Medical University, Wrocław, Poland; 3 Department of Histology and Embryology, Poznan University of Medical Sciences, Poznan, Poland; 4 Department of Pathomorphology and Oncological Cytology, Wroclaw Medical University, Wrocław, Poland; 5 Department and Clinic of Haematology, Blood Neoplasms and Bone Marrow Transplantation, Wroclaw Medical University, Wrocław, Poland

**Keywords:** D2-40, LYVE-1, VEGF-C, VEGF-D, Gastric cancer

## Abstract

**Background:**

One of the most important prognostic indicators in gastric cancer is the presence of metastases in lymph nodes. Even now, little is known about lymphangiogenesis in neoplastic tissue, and little is also known about the transmission of a neoplastic cell from the tumor mass into a lymphatic vessel.

**Methods:**

This study examined the relationships between the density of lymphatic vessels (LVD) stained immunohistochemically with lymphatic vessel endothelial hyaluronan receptor-1 (LYVE-1) and D2-40 (podoplanin) antibodies, the expression of vascular endothelial growth factor (VEGF)-C/D, selected clinical and pathomorphological factors, and the 5-year overall survival of gastric cancer patients.

**Results:**

Statistical analysis showed no impact of increased intratumoral or peritumoral LVD on gastric cancer patient survival, irrespective of the protein used to stain lymphatic vessels. Analysis showed that the probability of overall survival was decreased in the cases with enhanced VEGF-D immunoreactivity (*P* = 0.0045).

**Conclusion:**

The study showed that the studied markers cannot be used to determine the required extent of the surgical procedure, as they have no statistically significant correlation with the degree of progression of the cancer, the stage of the disease assessed according to the TNM 5th classification of malignant tumors, clinicopathological features, and patient survival. VEGF-D is the only marker that can be regarded as an unfavorable prognostic indicator for patients with advanced gastric cancer.

## Introduction

Gastric cancer (GC) is one of the most frequent cancers worldwide and is the second most common cause of cancer-related deaths [1]. Every year about 930,000 new cases are diagnosed [2]. Men are twice as likely as women to fall ill with GC. Asia has the highest GC incidence, and the lowest incidence is found in Australia, Africa, North America, southern Asia, and Oceania [3]. Despite the development of laparoscopic surgery techniques and the marketing of cancer-targeted therapies, advanced gastric cancer carries a highly unfavorable prognosis [4].

Gastric cancer is not common in Poland. Morbidity from GC is in fifth place for men and in seventh place for women for all cancers, and it is decreasing. Consequently, there is a lack of a prophylactic program for gastric cancer, and thus it is only recognized in its advanced stages.

At present the most important prognostic indicator for GC patients is the TNM classification, which describes the size of the primary tumor, the presence of metastases in lymph nodes, and distant metastases. Of key importance to improving treatment results, apart from an earlier detection of neoplastic changes, is the need to search for new prognostic indicators that would show with high probability the presence of metastases in lymph nodes and which would be helpful in establishing the extent of the required surgical procedure.

Lymphangiogenesis is the process of development of lymphatic vessels that occurs both during normal phenomena, e.g., during an inflammation or wound healing, and as part of many pathological processes, including carcinogenesis. A number of growth factors and their receptors are involved in lymphangiogenesis. It is directly initiated by vascular endothelial growth factors (VEGFs) C and D [5], which cause proliferation, migration, and formation of endothelial cells [6, 7].

VEGF-C expression is increased in many cancers, including gastric [8], esophageal [9], ovarian [10, 11], and breast [12] cancer. In GC a positive correlation has been shown between the VEGF-C level and metastases in lymph nodes [13]. Furthermore, Yonemura et al. [14] found that a high level of VEGF-C is an unfavorable prognostic factor.

VEGF-D induces both lymphangiogenesis and angiogenesis. An increased expression of VEGF-D is a negative prognostic factor, as is that of VEGF-C [15]. VEGF-C/D bind to specific vascular endothelial growth factor receptors (VEGFR). They have an affinity for VEGFR2, which is responsible for angiogenesis, and for VEGFR3, which until fairly recently was regarded as a lymphangiogenesis-specific receptor and is now also known to be present on vascular endothelium [16].

Immunohistochemical lymphatic vessel markers used in morphological diagnostics are LYVE-1 (lymphatic vessel endothelial hyaluronan receptor-1) and D2-40 (podoplanin) [17–19]. LYVE-1 is a hyaluronic acid receptor belonging to a receptor family that also includes CD44. LYVE-1 receptor expression has been found in lymphatic vessels in which VEGFR3 has also been detected. LYVE-1 have been discovered on both sides of lymphatic vessels, and so their likely function is the transmission of hyaluronic acid through cell membranes [20]. Furthermore, apart from its potential participation in endothelial cell metabolism, LYVE-1 plays a crucial role in the transfer of leukocytes through the lymphatic vascular wall [18, 20, 21]. D2-40 (podoplanin) plays a crucial role in preventing cellular adhesion and is strongly involved in the maintenance of glomerular permeability [22]. Studies have shown that D2-40 is a lymphatic vessel-specific glycoprotein and is not expressed on vascular endothelium. Podoplanin expression has been demonstrated also on osteocytes, podocytes, and follicular cells. In addition to lymphatic vessels, podoplanin immunoreactivity has been detected in kidney podocytes, osteoblastic cells, type I pneumocytes, and cells of the choroid plexus [23].

In this study, the density of lymphatic vessels measured by the expression of D2-40 and LYVE-1 and VEGF-C/D immunoreactivity in gastric cancer specimens was investigated by immunohistochemistry. The aim of the study was to find a correlation between TNM stage and clinicopathological features such as histopathological type, the presence of ulceration, the presence of an inflammatory infiltration, and angio- and lymphangio invasion and survival rate.

## Materials and methods

### Patients

The study was conducted on histopathological material obtained from patients operated for gastric cancer at the Second Department and Clinic of General and Oncological Surgery, Wroclaw Medical University, Poland in the years 1997–2006. A total of 42 consecutive patients were selected based on archival tissue and data concerning survival availability who underwent curative surgical treatment. The average age was 62 years; 31 were men and 11 women. Most of the cases were adenocarcinoma GIII. In the TNM scale most of the patients were in T2 or T3 (the same percentage of cases) N0 and M0 (5th edition of the TNM classification of malignant tumors), and most of the cases were advanced. It was typical for eastern Europe that gastric cancer, which is not common in our region, is recognized in the advanced stage. Every patient underwent total or subtotal gastrectomy with D2 lymphadenectomy. In all cases of total gastrectomy, splenectomy was also done with the open technique. The average number of lymph nodes was 19. Length of follow-up was 5 years (Table 1).

**Table 1:** Patient and tumor characteristics

Feature	No.
All patients	42
Age in years (mean, 62)	
<60	19
>60	23
Gender	
Female	11
Male	31
TNM, T feature	
T1	1
T2	19
T3	19
T4	3
TNM, N feature	
N0	24
N1	12
N2	6
N3	0
TNM, M feature	
M0	28
M1	14
Histopathological tumors characteristics	
Grade 1	0
Grade 2	15
Grade 3	27
Presence of ulceration	29 cases
Presence of inflammation	33 cases
Lymphatic vessels cancer thrombosis	4 cases
Disease-free 5-year survival	16 cases

The study was retrospective and had the approval of the special ethical committee of Medical University of Wrocław (10.2006).

### Tissue specimens

The tissue samples were fixed in 10 % buffered formalin and embedded in paraffin. In each case, hematoxylin and eosin-stained slides were subjected to histopathological evaluation by two pathologists. Analyzed clinicopathological data were as follows: TNM stage, histopathological type, presence of angio- and lymphangio invasion, ulceration, inflammation and long-term survival rate.

### Immunohistochemistry

Immunohistochemical analysis were conducted at the Laboratory of the Department of Pathomorphology in Lower Silesian Oncology Centre in Wroclaw, Poland. Formalin-fixed, paraffin-embedded tissue was freshly cut into 5-μm sections. The sections were mounted on Superfrost slides (Menzel Gläser, Germany), dewaxed with xylene, and gradually hydrated. Activity of endogenous peroxidase was blocked by 5-min exposure to 3 % H_2_O_2_. All the studied sections were boiled for 15 min at 250 W in Antigen Retrieval Solution (DakoCytomation, Denmark). Then, immunohistochemical reactions were performed using (1) rabbit polyclonal antibodies detecting LYVE-1 (dilution 1:100; RELIATech, Germany); (2) mouse monoclonal antibodies detecting D2-40 (dilution 1:100; DakoCytomation, Denmark); (3) mouse monoclonal antibodies detecting VEGF-C (dilution 1:150; R&D Systems, USA); (4) mouse monoclonal antibodies detecting VEGF-D (dilution 1:150; R&D Systems). Tested sections were incubated with antibodies for 1 h at room temperature. Subsequent incubations involved biotinylated antibodies (15 min, room temperature) and streptavidin–biotinylated peroxidase complex (15 min, room temperature) (LSAB+, HRP; DakoCytomation, Denmark). NovaRed (Vector Laboratories, UK) was used as a chromogen (10 min, at room temperature). All the sections were counterstained with Meyer’s hematoxylin. The control group for the antibodies studied was composed of healthy stomach specimens.

### Immunopathomorphological evaluation

The expression of the studied proteins was assessed under a microscope (Olumpus BX41) using computer microscopic image analysis (software: AnalySIS DOCU, ver. 3.2 for Windows 95/98/NT; Soft Imaging, licence 100 7557). All the preparations were examined by two pathologists, who performed independently immunopathomorphological evaluation based on analysis of large tissue slides (about 2 cm in diameter). Because of different scoring fields some discrepancies between LVD occurred (as documented on microphotographs), and these cases were excluded from the analysis.

LYVE-1 and D2-40 protein expression was evaluated using a quantitative method: a modified Weidner’s method [24]. Vessel density was evaluated within neoplastic tissue and within healthy tissue outside the tumor. A microscopic image from one preparation magnified 200 times was transferred to a computer program, which automatically counted all positively stained vessels from three random fields of view with the highest density. Each individual vessel or cluster of endothelial cells was regarded as a hot point and was counted as one microvessel. Afterward, the average number of vessels from the three fields of view was calculated.

The expression of VEGF-C and VEGF-D proteins was calculated using a semiquantitative method. Two immunohistochemical reaction parameters were considered when evaluating the expression of the foregoing proteins: the percentage of cells with a positive cytoplasmic reaction (the percentage of reactive tissue) and the intensity of a reaction. The ultimate immunohistochemical reaction results are expressed in the semiquantitative IRS (Immuno Reactive Score) scale according to Remmele and Stenger [25]. The scale assigns a score for a percentage of cells demonstrating reaction (0–4 points) and for the intensity of a reaction (0–3 points). The final result is the product of the scores for the parameters analyzed (0–12 points) and is referred to as an IRS factor (Table 2).

**Table 2:** Evaluation of vascular endothelial growth factor (VEGF) expression

Percentage of positive cells	Points	Intensity of reaction	Points
IRS (Immuno Reactive Score) modified by authors^a^			
No positive cells	0	No reaction	0
<25 % positive cells	1	Weak color reaction	1
25–50 % positive cells	2	Moderate intensity	2
51–75 % positive cells	3	Intense reaction	3
>75 % positive cells	4		

^a^IRS score (Immuno Reactive Score) according to Remmele and Stenger [25], modified by the authors

### Statistical analysis

The conformity of distribution of individual variables with the standard normal distribution was checked by means of the Shapiro–Wilk test, the homogeneity of variances was verified with Levene’s test, the significance of differences in means between two variables with a normal distribution was verified with a *t* test, and for analysis of variance in groups of three or more variables an *F* test was used (ANOVA). Variances between variables with a distribution significantly differing from a normal one was verified by means of the Mann–Whitney *U* test (where two variables were compared) or the Kruskal–Wallis test (where there were more than two variables). The relationship between two variables with a normal distribution was analyzed using Spearman’s correlation. Dependencies between variables with a distribution other than normal were analyzed by means of Spearman’s or a gamma correlation. The correlation between the time of survival and individual stained antibodies was investigated with a Cox regression analysis.

## Results

### Density and distribution of lymphatic vessels stained with D2-40 and LYVE-1 markers

**Fig. 1:**
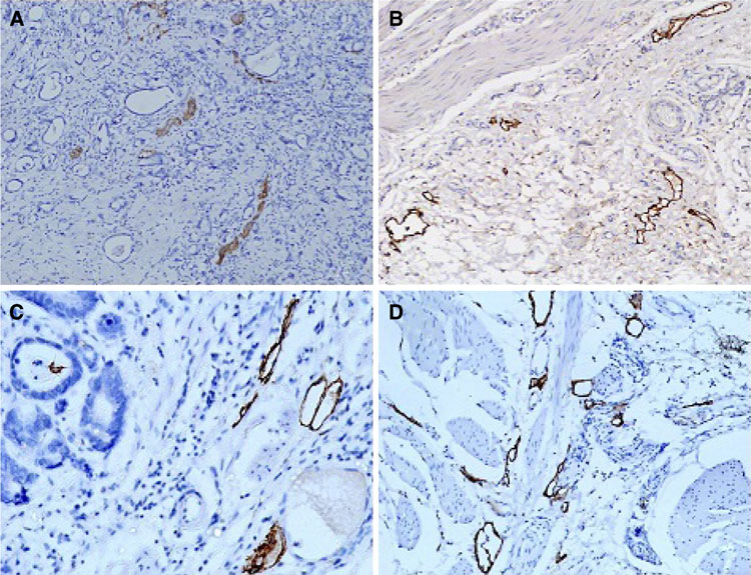
Lymphatic vessel density stained with D2-40 (podoplanin) and lymphatic vessel endothelial hyaluronan receptor-1 (LYVE-1). Expression of D2-40 in intratumoral compartment (**a**) and peritumoral zone (**b**); expression of LYVE-1 in intratumoral area (**c**) and peritumoral zone (**d**).** a**,** b** ×100;** c**,** d** ×200

Lymphatic vessels stained with D2-40 marker were visible throughout neoplastic tissue without a distinct borderline. The mean LVD (lymph vessel density) in neoplastic tissue within the field of view was 6.5 (minimum, 0; maximum, 21.7) (Fig. 1a). Podoplanin-stained lymphatic vessels were also found in the peritumoral zone. Their density there was statistically significantly higher (Wilcoxon test: *Z* = 3.4, *P* = 0.0007) than that within the tumor, and on average amounted to 8.8 vessels within one field of view. The smallest number of vessels for this range was 1.1 and the largest 23.1 (Fig. 1b).

Interestingly, lymphatic vessels stained with LYVE-1 were mostly observed in the peritumoral zone. The mean intratumoral LVD within a field of view was 1.4, with LVD varying between 0 and 10 (Fig. 1c). In the peritumoral area the mean LYVE-1-stained LVD was higher than that within the tumor, 6.7 within the field of view, and the density difference between the compartments was statistically significant (Wilcoxon test: *Z* = 4.86, *P* = 0.000001). The number of vessels within the field of view ranged between 0 and 23 (Fig. 1d).

### Comparison of specificity of D2-40 and LYVE-1 markers in respect of lymphatic vessels

**Fig. 2:**
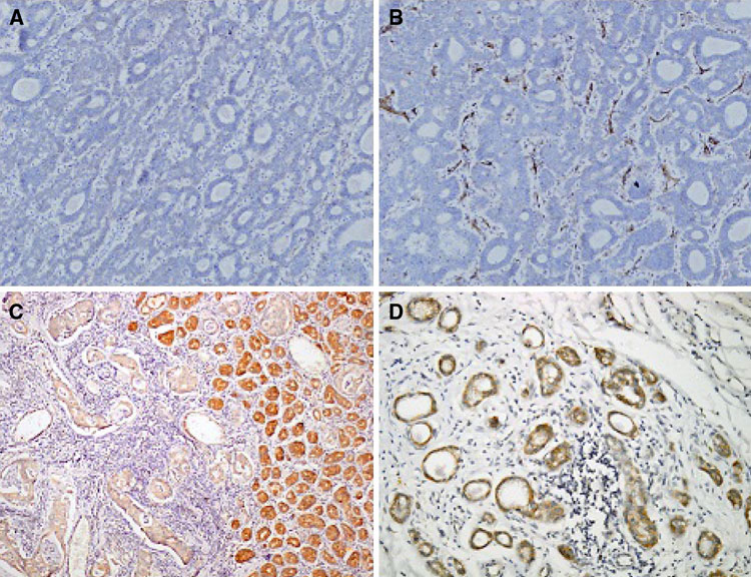
Comparison of lymphatic vessels staining with two different markers in pairs in the same case. Intratumoral expression of LYVE-1 (**a**), D2-40 (**b**), vascular endothelial growth factors (VEGF-C) (**c**), and VEGF-D (**d**).** a**,** b** ×200;** c**,** d** ×100

Comparison of the densities of lymphatic vessels stained with the two markers demonstrated that both the mean and maximum LVD were higher if D2-40 had been used (Fig. 2a, b). Analysis revealed that the difference was statistically significant both in the intratumoral compartment and in the peritumoral zone (Wilcoxon test: *Z* = 4.9, *P* = 0.000001; *Z* = 2.28, *P* = 0.02, respectively).

### Expression of VEGF-C and VEGF-D in GC and their correlation with LVD

The mean IRS of VEGF-C was 3 (Fig. 2c) and was 0.8 for VEGF-D 0.8 (Fig. 2d). A positive correlation between the expression of both markers in neoplastic tissue was found (*P* = 0.001). No statistically significant correlation between VEGF-C/D immunoexpression and the density of lymphatic vessels stained with LYVE-1 or D2-40 was observed in any of the studied compartments except for the correlation between VEGF-C and also VEGF-D and intratumoral LVD marked with LYVE-1 (Table 3).

**Table 3:** Correlations between VEGF-C/D and lymphatic vessel density measured by lymphatic vessel endothelial hyaluronan receptor-1 (LYVE-1) and D2-40 (podoplanin) in intratumoral and peritumoral compartments

	LYVE-1 intratumoral LVD		LYVE-1 peritumoral LVD		D2-40 intratumoral LVD		D2-40 peritumoral LVD	
	*r*	*P*	*r*	*P*	*r*	*P*	*r*	*P*
VEGF-C	0.25	0.021	0.19	0.08	0.2	0.06	0.18	0.08
VEGF-D	0.21	0.04	−0.01	0.9	0.18	0.5	−0.07	0.5

*r* correlation ratio, *P* statistical validity, *LVD* lymphatic vessels density

An analysis of correlation between TNM classification parameters, the expression of VEGF-C/D growth factors, and the density of LYVE-1 and D2-40 stained lymphatic vessels was carried out. No statistically significant dependence between the depth of the cancerous infiltration, the foci of nodal metastasis and the presence of distant metastases, and the analyzed immunohistochemical and pathomorphological parameters of proteins was demonstrated. One significant correlation was noticed: between the presence of distant metastases and enhanced immunoreactivity of VEGF-C in the neoplastic compartment (Table 4).

**Table 4:** Correlation between expression of VEGF-C/D, lymphatic vessel density measured by LYVE-1 and D2-40 in peritumoral and intratumoral compartments, and TNM parameters

	T (*P*)	N (*P*)	M (*P*)
LYVE-1 intratumoral LVD	0.1238	0.7772	0.1533
LYVE-1 peritumoral LVD	0.1879	0.6509	0.8604
D2-40 intratumoral LVD	0.1873	0.2829	0.3337
D2-40 peritumoral LVD	0.3543	0.3027	0.3677
VEGF-C	0.8415	0.2712	0.0176
VEGF-D	0.2176	0.7473	0.3337

*P* statistical validity, *LVD* lymphatic vessel density

Furthermore, the correlations between the expression of growth factors VEGF-C/D, the density of LYVE-1- and D2-40-stained lymphatic vessels, and the clinical and pathomorphological factors of GC, such as histopathological type, presence of ulceration, the presence of an inflammatory infiltration and angio- and lymphangio invasion, investigated with the Kruskall–Wallis test or the Mann–Whitney test, were statistically nonsignificant.

**Fig. 3:**
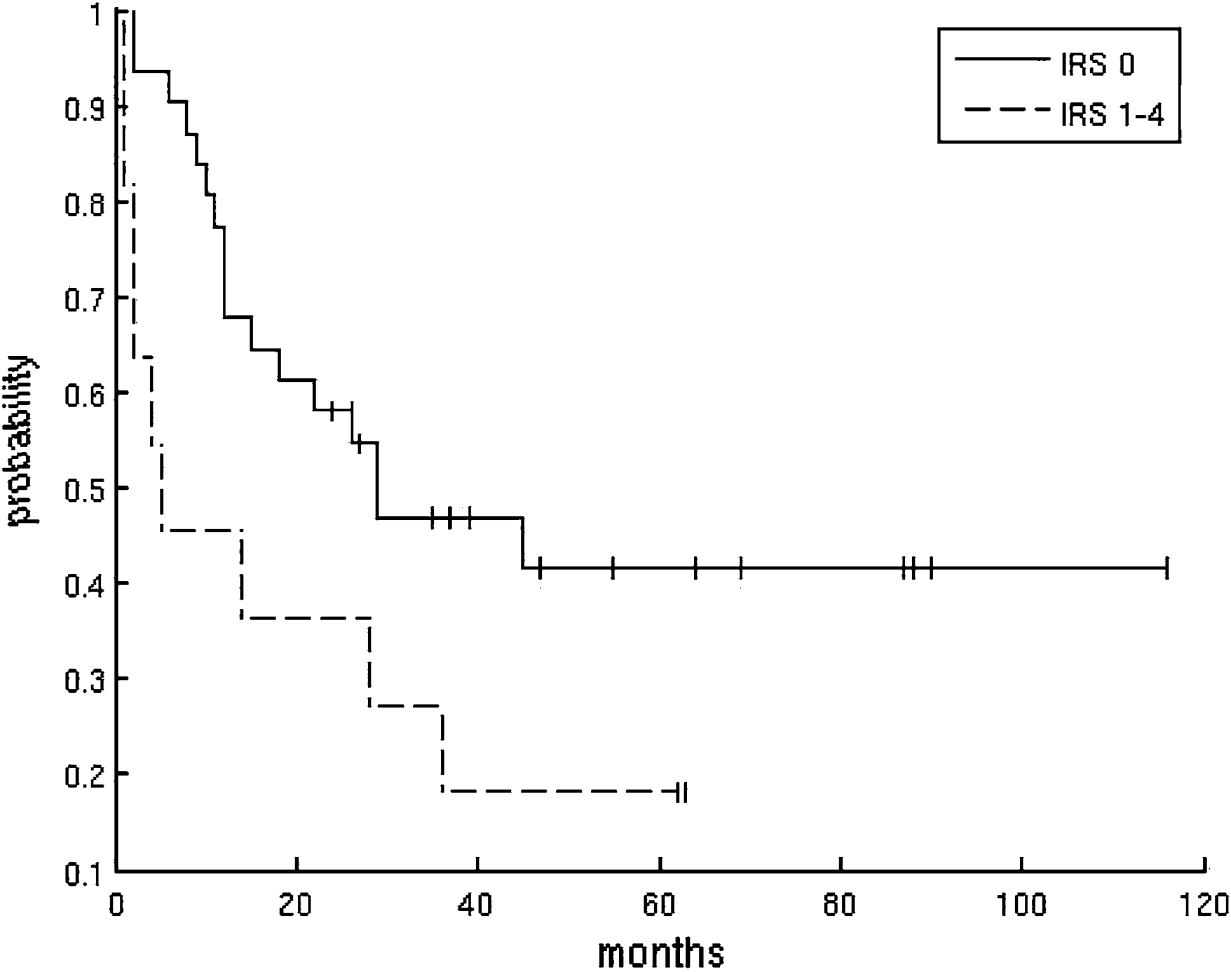
Kaplan–Meier curve for two groups of patients: *solid line*, VEGF-D expression (Immuno Reactive Score, *IRS*) = 0;* dashed line* *IRS* = 1–4)

A Cox regression analysis has shown no impact of increased intratumoral or peritumoral LVD on GC patient survival, irrespective of the type of markers. A similar relationship was found for VEGF-C protein. However, the study indicated a statistically significant correlation between a high level of VEGF-D and patients’ long-term survival, with a significance of *P* = 0.0045 (according to Cox regression analysis). Patients were divided into two separate groups: the first consists of patients whose expression of VEGF-D proteins was equal to 0 on the IRS scale (31 patients), whereas the second group included all cases with VEGF-D expression between 1 and 4 on the IRS scale (11 patients). For each group a Kaplan–Meier curve was calculated (Fig. 3). Based on statistical analysis, the survival rate within the first group could be estimated at around 41 %, whereas within the second group the chance for remaining alive is estimated as around 18 %. It is worth pointing out that both curves were tested using the log-rank test with the null hypothesis, which considered both groups had identical survival functions. The test’s result (*Z* = −2.03948, *P* = 0.0207) forced rejection of the null hypothesis. Therefore, we can state that there is a significant difference in survival rate depending on whether the patient has VEGF-D expression equal to 0 or more than 0 (1–4). Based on the calculations, it could be stated that patients with a positive VEGF-D expression level have a chance of surviving treatment more than twofold less than do the patients with IRS equal to 0.

## Discussion

In this study, the density of lymphatic vessels was measured by the expression of D2-40 and LYVE-1 and VEGF-C/D immunoreactivity in gastric cancer specimens was investigated by immunohistochemistry: the correlation between the expression parameters of these proteins and clinicopathological features was statistically analyzed.

The most interesting surgical issue was the connection between the immunohistochemical markers studied and the presence of metastases in lymph nodes (N). The study did not reveal any correlation between the N feature and the density of lymphatic vessels stained with either LYVE-1 or D2-40. The lack of any correlation between metastases in lymph nodes and the density of lymphatic vessels stained with either marker indicates their limited usefulness as a marker of the presence of neoplastic cells in lymph nodes. Consequently, LVD cannot be used to determine the probability of the existence of metastases in lymph nodes and thus is not useful when deciding whether to perform supplementary lymphadenectomy. However, some studies have confirmed a connection between lymphangiogenesis and the existence of metastases in lymph nodes and have found LVD to be an independent prognostic factor [26, 27].

Further, our study failed to reveal any statistically significant correlation between the analyzed clinicopathological parameters and the expression of VEGF-C/D growth factors. Numerous studies have confirmed the relationships between VEGF-C levels and the presence of metastases in lymph nodes [3, 28, 29]. However, there are studies in which no such correlation has been found [30]. The VEGF-C factor is connected with both angiogenesis and lymphangiogenesis pathways. Despite its association with the two processes, many studies have detected a close correlation between VEGF-D levels and the presence of metastases in lymph nodes [29, 31]. This study failed to detect any connection between VEGF-D and the presence of nodal metastases.

The last aspect under study was the presence of distant metastases (M). Only VEGF-C and the presence of distant metastatic changes was demonstrated. Consequently, this is the only factor under study that can be regarded as an organ progression factor for GC.

Another issue under consideration as part of the study was the relationship between lymphangiogenesis and patient survival. The available literature does not agree on the existence of a connection between LVD and patient survival. According to Nakamura and Kakudo [27], there is a correlation between the density of lymphatic vessels stained with podoplanin and patient survival and prognosis. However, there are studies indicating no connection between LVD and patient survival in gastric cancer [29]. The discrepancy between results obtained by various authors attests to the need to conduct further studies into lymphangiogenesis and its influence on the development and progression of cancer. Similarly, the issue of VEGF-C as a prognostic factor seems to be unresolved. This study showed no association between VEGF-C and patient long-term survival, similar to other publications [15, 32]. However, there are reports about a correlation between increased VEGF-C levels and a shorter survival and a worse prognosis [14]. In the case of VEGF-D, as in the case of VEGF-C, contradictory results concerning the prognostic role of the protein in the neoplastic process have been obtained in various studies. No correlation with survival in non-small cell lung cancer was observed [33]. Some studies, however, have indicated a close relationship between VEGF-D and long-term survival, with VEGF-D functioning as a prognostic factor [15]. This study showed that the growth factor VEGF-D is inversely correlated with the survival of GC patients and can be regarded as a helpful prognostic factor.

With a view to finding an answer to the question whether lymphangiogenesis is in any way linked to the clinicopathological features of the neoplasm, such as histopathological type, presence of an inflammatory reaction, ulceration, or cancer cell thrombosis, as an indicator of GC progress, a number of analyses were carried out. This study found no correlation with the density of lymphatic vessels stained with LYVE-1 and D2-40, growth factors VEGF-C/D, and any of the clinicopathological features such as histopathological type of GC, presence of ulceration, inflammatory infiltration, or tumor embolism in blood or lymphatic vessels.

This study was limited by the small number of cases, only 42. In the period of the 10 years observed, more than 42 cases underwent surgery in the 2nd Department of General and Oncological Surgery in Medical University of Wrocław. The average number of gastric cancer cases is about 50 per year, but some of the operated cases were palliative efforts, not curative. The study was retrospective, and the data in the documentation were not complete (including survival rate, histopathological examination, or such information as address). Another point is that the patients often were not recruited from our region of Lower Silesia and it was impossible to obtain data such as survival rate. Although the number of the cases is too small to draw a conclusion, it is worth mentioning especially when it impacts advanced cases, not the early stages, which are characteristic for the eastern countries.

The results yielded by the study call for a further investigation into the lymphangiogenesis pathway occurring during carcinogenesis as there are many ambiguities. The process may differ from that taking place in healthy tissue and may be subject to modification by other factors. Lymphatic vessels created as part of the neoplastic process are probably structurally different from physiological vessels. Any links between individual stages of the lymphangiogenesis pathway seem complicated and are still relatively unknown, and the results of many studies remain inconsistent. An in-depth knowledge of the process could offer GC patients a chance of longer survival. Currently, remedies are being sought to slow down cancer metastasis to lymph nodes, which is of paramount importance in the case of gastric cancer. Studies conducted on tumor tissue models attest to the clinically satisfactory effectiveness of drugs that slow down lymphangiogenesis by inhibiting VEGF-R3, the receptor that is the most specific to lymphangiogenesis [31, 34].

## References

[1] Parkin DM, Bray F, Ferlay J, Pisani P (2005). Global cancer statistics, 2002. Cancer J Clin.

[2] Matsubara J, Yamada Y, Nakajima TE, Kato K, Hamaguchi T, Shirao K (2008). Clinical significance insulin-like growth factor type 1 receptor and epidermal growth factor receptor in patients with advanced gastric cancer. Oncology.

[3] Amioka T, Kitadai Y, Tanaka S (2002). Vascular endothelial growth factor C expression predicts lymph node metastasis of human gastric carcinoma invading the submucosa. Eur J Cancer.

[4] Bang YJ, Van Cutsem E, Feyereislova A, Chung HC, Shen L, Sawaki A, Lordick F (2010). Trastuzumab in combination with chemotherapy versus chemotherapy alone for treatment of HER2-positive advanced gastric or gastro-oesophageal junction cancer (ToGA): a phase 3, open-label, randomized controlled trial. Lancet.

[5] Yonemura Y, Fushida S, Bando E, Kinoshita K, Miwa K, Endo Y (2001). T: lymphangiogenesis and the vascular endothelial growth factor receptor (VEGFR3) 3 in gastric cancer. Eur J Cancer.

[6] Ferrara N, Gerber H-P, Le Couter J (2003). The biology of VEGF and its receptors. Nat Med.

[7] Hirakawa S, Brown LF, Kodama S (2007). VEGF-C-induced lymphangiogenesis in sentinel lymph nodes promotes tumor metastasis to distant sites. Blood.

[8] Gou HF, Chen XC, Zhu J, Jiang M, Yang Y, Cao D (2011). Expression of Coc-2 and VEGF-C in gastric cancer: correlations with lymphangiogenesis and prognostic implications. J Exp Clin Cancer Res.

[9] Kozlowski M, Naumnik W, Niklinski J, Milewski R, Dziegielewski P, Laudanski J (2011). Vascular endothelial growth factor C and D expression correlates with lymph node metastasis and poor prognosis in patients with resected esophageal cancer. Neoplasma.

[10] Huang KJ, Sui LH. The relevance and role of vascular endothelial growth factor C, matrix metalloproteinase-2 and E-cadherin in epithelial ovarian cancer. Med Oncol 2012;29(1):318–23. doi: 10.1007/s12032-010-9817-4. 10.1007/s12032-010-9817-4 21264536

[11] Sinn V, Darb-Esfahni S, Wirtz RM, Faggad A, Wechert W, Buckendahl AC (2009). Vascular endothelial growth factor C mRNA expression is a prognostic factor in epithelial ovarian cancer as detected by kinetic RT-PCR in formalin-fixed paraffin-embedded tissue. Virchows Arch.

[12] Nakamura Y, Yasuoka H, Tsujimoto M, Imabun S, Nakahara M, Nakako H (2005). Lymph vessel density correlates with nodal status, VEGF-C expression and prognosis in breast cancer. Breast Cancer Res Treat.

[13] Liu X, Sun XD, Wu JM (2004). Expression and significance of VEGF C and FLT-4 in gastric cancer. World J Gastroenterol.

[14] Yonemura Y, Endo Y, Fujita H (1999). Role of vascular endothelial growth factor C expression in development of lymph node metastasis in gastric cancer. Clin Cancer Res.

[15] Jutter S, Wibman C, Jons T (2006). Vascular endothelial growth factor D and its receptor VEGFR3: two novel independent prognostic markers in gastric adenocarcinoma. J Clin Oncol.

[16] Ferrara N, Davis-Smyth T (1997). The biology of vascular endothelial growth factor. Endocr Rev.

[17] Coskun U, Yamac D (2009). Peritumoral lymphatic microvessel density associated with tumor progression and poor prognosis in gastric carcinoma. J Surg Res.

[18] Jackson DG, Prevo R, Clasper S, Banejri S (2001). LYVE-1, the lymphatic system and tumor lymphangiogenesis. Trends Immunol.

[19] Zeng Y, Opeskin K, Horvath LG (2005). Lymphatic vessel density and lymph node metastasis in prostate cancer. Prostate.

[20] Gordon EJ, Gale NW, Harvey NL (2008). Expression of the hyaluronan receptor LYVE-1 is not restricted to the lymphatic vasculature: LYVE-1 is also expressed on embryonic blood vessels. Dev Dyn.

[21] Jackson DG (2004). Biology of the lymphatic marker LYVE-1 and applications in research into lymphatic trafficking and lymphangiogenesis. Acta Pathol Microbiol Immunol Scand.

[22] Raica M, Cimpean AM, Ribatti D (2008). The role of podoplanin in tumor progression and metastasis. Anticancer Res.

[23] Wicki A, Christofori G (2007). The potential role of podoplanin in tumor invasion. Br J Cancer.

[24] Weidner N, Semple J, Welich W, Folkman J (1991). Tumor angiogenesis and metastasis: correlation in invasive breast carcinoma. N Engl J Med.

[25] Remmele W, Stenger HE (1987). Vorschlag zur einheitlicchen Definition eines Immunreaktiven Score (IRS) fuer den immunohistochemischen Oestrogenrezeptor-Nachweis (ER-ICA) im Mammarkarzinomgewebe. Pathologe.

[26] Fujimoto A, Ishikawa Y, Ishii T (2001). Significance of lymphatic invasion on regional lymph node metastasis in early gastric cancer using LYVE-1. Immunohistochemical analysis. Am J Clin Pathol.

[27] Nakamura KK (2006). Importance of lymph vessels in gastric cancer: a prognostic indicator in general and a predictor for lymph node metastasis in early stage cancer. J Clin Pathol.

[28] Gao P, Zhou GY, Zhang QH, Xiang L, Zhang SL, Li C, Sun YL (2008). Clinicopathological significance of peritumoral lymphatic vessel density in gastric carcinoma. Cancer Lett.

[29] Coskun U, Yamac D (2009). Peritumoral lymphatic microvessel density associated with tumor progression and poor prognosis in gastric carcinoma. J Surg Res.

[30] Hye U, Oh YH, Park YW (2008). Correlation of vascular endothelial growth factor-D expression and VEGFR 3-positive vessel density with lymph node metastasis in gastric carcinoma. J Korean Med.

[31] Yashiro M, Shinto O, Nakamura K, Tendo M, Matsuoka T, Matsuzaki T, Kaizaki R, Ohira M, Miwa A, Hirakawa K (2009). Effects of VEGFR-3 phosphorylation inhibitor on lymph node metastasis in an orthotopic diffuse-type gastric carcinoma model. Br J Cancer.

[32] Ichikura T, Tomimatsu S, Ohkura E (2001). Prognostic significance of the expression of vascular endothelial growth factor and VEGF C in gastric carcinoma. J Surg Oncol.

[33] Ko YH, Jung CK, Lee MA, Byun JH, Kang JH, Lee KY, Jo KH, Wang YP, Hong YS (2008). Clinical significance of vascular endothelial growth factors (VEGF)-C and -D in resected non-small cell lung cancer. Cancer Res Treat.

[34] Shimizu K, Kubo H, Yamaguchi K (2004). Suppression of VEGFR3 signaling inhibits lymph node metastasis In gastric cancer. Cancer Sci.

